# The Potent Anti-Tumor Effects of Rhodiola Drinking Are Associated with the Inhibition of the mTOR Pathway and Modification of Tumor Metabolism in the UPII-Mutant Ha-Ras Model

**DOI:** 10.3390/cancers15123086

**Published:** 2023-06-07

**Authors:** Zhongbo Liu, Noriko N Yokoyama, Liankun Song, Jun Xie, Zhina Sadeghi, Yi Xi Wu, Sarah Yee, Xue-Ru Wu, Beverly Wang, Edward Uchio, Xiaolin Zi

**Affiliations:** 1 Department of Urology, University of California, Irvine, CA 92868, USA; liuzb727@gmail.com (Z.L.); noriko_yok@hotmail.com (N.N.Y.); liankuns@hs.uci.edu (L.S.); xiej@hs.uci.edu (J.X.); zhinas@hs.uci.edu (Z.S.); yxwu@hs.uci.edu (Y.X.W.); euchio@hs.uci.edu (E.U.); 2 Veterans Affairs New York Harbor Healthcare System, New York, NY 10010, USA; xue-ru.wu@nyulangone.org; 3 Department of Pathology and Laboratory Medicine, University of California, Irvine, CA 92868, USA; bevwang@hs.uci.edu; 4 Chao Family Comprehensive Cancer Center, University of California, Irvine, CA 92868, USA; 5 Veterans Affairs Long Beach Healthcare System, Long Beach, CA 90822, USA

**Keywords:** rhodiola, bladder cancer chemoprevention, mTOR, metabolism

## Abstract

**Simple Summary:**

Bladder cancer occurs mainly in older people and is expensive to manage. Swedish Herbal Rhodiola-5 (SHR-5), a standardized *Rhodiola Rosea* extract from the Swedish Herbal Institute (the SHI, Gothenburg, Sweden), has been in clinical studies for managing mild to moderate depression and physical and mental fatigue. Previous studies have shown that SHR-5 extended the life span of the fruit fly through a mechanism that was not associated with dietary restriction. In this study, we examined whether drinking SHR-5 can delay tumor development in a superficial bladder cancer mouse model. Drinking SHR-5 daily markedly improved the survival rate of the bladder tumor-bearing mice, by an absolute 61.7%, and reduced urinary tract obstruction and tumor burden. SHR-5 drinking reshaped the tumor metabolism via the inhibition of the mTOR leading to a decreased tumor cell proliferation.

**Abstract:**

**Background:** SHR-5 has been used as an “adaptogen” for enhancing physical and mental performance and for fighting stress in the healthy population. The purpose of this study is to determine the chemopreventive efficacy of SHR-5 for superficial bladder cancer and to investigate the underlying mechanisms of action. **Methods:** UPII-mutant Ha-ras bladder-cancer-transgenic mice, that developed low-grade and noninvasive papillary transitional urothelial cell carcinoma, were fed with 1.25 and 6.25 mg/mL SHR-5 in drinking water for 6 months. The survival of the mice, obstructive uropathy, tumor burden and morphology, and proliferation were evaluated by pathological, molecular, metabolic, and statistical analyses. **Results:** Approximately 95% or more of the male UPII-mutant Ha-ras mice that drank SHR-5 daily survived over 6 months of age, while only 33.3% of those mice that drank normal water survived over 6 months of age (*p* < 0.0001); SHR-5 drinking exposure also reduced tumor-bearing bladder weight and urinary tract obstruction and inhibited mTOR signaling in neoplastic tissues. Global metabolic analysis revealed that SHR-5 resulted in increased phenolic metabolites and decreased CoA, a critical metabolic cofactor for lipid metabolism. **Conclusions:** Our findings highlight the potential of SHR-5 as an anti-aging agent for bladder cancer prevention through reshaping tumor metabolism via the inhibition of the mTOR signaling. Global metabolomics profiling provides a unique and efficient tool for studying the mechanisms of complex herb extracts’ action.

## 1. Introduction

The incidence of human urinary bladder cancer increases markedly with age, suggesting a mechanistic connection between aging and bladder carcinogenesis, and the potential use of anti-aging agents in bladder cancer chemoprevention [1]. Aging is a major risk factor for many common cancers, including bladder cancer. More than 71% of patients are older than 65 years at the first diagnosis of bladder cancer [1]. As the population ages, one can expect an increasing burden of bladder cancer in the coming years. Since cancer and aging are both associated at the molecular level, anti-aging agents could be ideal candidates for cancer prevention in the elderly. However, due to the complexity of aging, currently, there are very few agents with anti-aging effects. 

*Rhodiola rosea L* (*R. rosea*), also known as “golden root”, is a perennial herbaceous plant of the Crassulaceae family, widely distributed at high altitudes (up to 2280 m) in the arctic and mountainous regions throughout Europe and Asia [2]. *R. rosea* has a history of centuries of folk uses, mainly in enhancing both the physical and mental performance in healthy populations [3]. The pharmacological and medicinal properties of rhodiola are species-dependent [4]. *R. rosea* has been reported to have anti-aging, anti-hypoxia, anti-stress, anti-cancer, anti-inflammatory, and antioxidative functions [3,4]. There are several studies demonstrating the anticancer activities of *R. rosea* extracts against liver tumors, breast cancer cells, and others [5,6,7]. Anecdotal evidence from a study in Russia, involving 12 patients with superficial bladder cancer, showed that an *R. rosea* extract decreased the average frequency of cancer relapse by half [8].

SHR-5, a standardized *R. rosea* extract, with active components of about 3 percent rosavins and 0.8–1 percent salidroside, was manufactured, according to good manufacturing practice, by the Swedish Herbal Institute (the SHI, Gothenburg, Sweden). SHR-5 is the only preparation from *R. rosea* that has passed extensive toxicological studies and has been certified safe for both animals and humans [9]. We have previously demonstrated that SHR-5 and one of its bioactive components, salidroside, inhibit the growth of bladder cancer cell lines with minimal effect on the growth of non-malignant bladder urothelial cells via the inhibition of the mTOR pathway and induction of autophagy [10]. Udintsev et al. [11] described how the phenol components of *Rhodiola Rosea* extracts inhibited the tumor cells of mice with Ehrlich ascites cancer but stimulated the functional activity of bone marrow stem cells. Chen et al. [12] reported that salidroside, a main component of *Rhodiola Rosea* extracts, protects against doxorubicin-induced cardiomyopathy by activating the AMPK pathway leading to a marked down-regulation of ferroptosis cell death. These results indicate that Rhodiola exerts anti-tumor effects while protecting normal cells and functions.

Here, we examined whether SHR-5 drinking could prevent the development of papillary urothelial cell carcinoma (UCC) or delay their progression in vivo in the UPII-mutant Ha-ras transgenic model. We have demonstrated that SHR-5 drinking markedly reduces the tumor burden and extends the survival of urothelial tumor-bearing mice, as well as preventing or delaying the occurrences of papillary UCC. The in vivo mechanisms of SHR-5’s action are associated with anti-proliferation through the rewiring of tumor cell metabolism via the inhibition of the mTOR pathway.

## 2. Materials and Methods

### 2.1. Mouse Breeding, Southern Blotting, and Genotyping 

**Figure 1:**
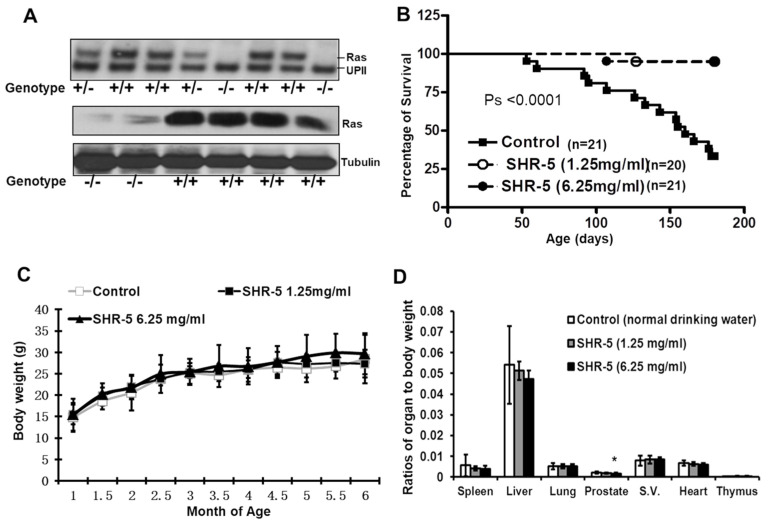
SHR-5 drinking increases the survival of the UPII-Ha-ras transgenic mice. (**A**) Upper panel: genotyping by Southern blotting analysis of NcoI-digested mouse tail DNAs shows wild-type mice with the endogenous UPII fragment, heterozygous mice with Ras/UPII ratio of about 1:2, and homozygous mice with Ras/UPII ratio of about 1:1. Lower panel: Western blotting analysis of Ras expression in the urothelial tissues of wild-type and homozygous mice. (**B**) Survival curves of male homozygous UPII-mutant Ha-ras transgenic mice that drank regular water (vehicle control) or 1.25 or 6.25 mg/mL SHR-5-supplemented water until 180 days of age. (**C**) The mean body weight was documented every two weeks. (**D**) Ratios of organ weights to body weights at the end of the experiment of male UPII-mutant Ha-ras mice fed with indicated drinks as shown in the figure. Error bars: standard deviation. “*” indicates *p <* 0.05.

Homozygous male UPII-mutant Ha-ras transgenic mice were selected for the experimental protocol. Wild-type or nontransgenic types were utilized in safety studies or as controls. Mouse breeding, Southern blotting, and genotyping were performed as described in our previous publications [13,14]. Homozygous UPII-mutant Ha-ras mice have a 1:1 ratio of the mutant human Ha-ras gene to the endogenous UPII gene, and express high levels of Ras protein in bladder tissues as shown in Figure 1A.

### 2.2. Experimental Animal Groups, Treatment, and Necropsy

The *R. rosea* extract (SHR-5) was obtained from the Swedish Herbal Institute (Göteborg, Sweden) according to good manufacturing practice [15]. Amounts of 125 mg and 625 mg SHR-5 were freshly dissolved in 100 mL distilled water and put into a light protected water bottle for mice to drink freely. The water was changed every two days. Four-week-old, genotyped homozygous UPII-mutant Ha-ras male transgenic mice (*n* = 20–21) were fed with regular water or water with 1.25 or 6.25 mg/mL SHR-5, and with standard AIN93M diet, and then sacrificed at the age of 180 days. Mice were permitted free access to food and water. All animals were examined daily for morbidity, mortality, clinical signs of ataxia, and toxicological effects including respiratory depression, neurobehavioral abnormalities, color of skin and eyes (a sign of liver toxicity), and motor activity. Food consumption and animal body weight were recorded bi-weekly. Animal care and treatments were performed according to institutional guidelines and the approved protocol by UCI (protocol #:2004-2540).

At the end of treatment, all mice were euthanized by CO2 asphyxiation. Blood samples were collected by cardiac puncture and plasma was separated by centrifugation. Urine samples were obtained by bladder massage. A laparotomy was performed to expose all major organs which were inspected for frank toxicity and any visible abnormality. Photographs were taken with a digital camera to document the animals and their urogenital systems in situ. All non-bladder organs were removed, weighted, and fixed in formalin for standard hematoxylin and eosin (H&E)-stained slide preparation and examination. Any evidence of edema, abnormal organ size, or change in appearance of non-bladder organs was noted. A portion of the urinary bladder was fixed in 10% neutral-buffered formalin for histopathological evaluation and the rest was snap-frozen in liquid nitrogen and stored at −80 °C for further analysis. Histological lesions of each urinary bladder were evaluated by a pathologist (B.W.) blinded to the experimental groups and categorized into simple hyperplasia, papillary hyperplasia, nodular hyperplasia, papilloma, and papillary UCC, as detailed in our previous publications [13,14,16].

### 2.3. Urine Analysis

The urine of each mouse, at four months of age, was collected through bladder massage and tested with Chemstrip 4MD urinalysis test strips (Roche Diagnostics, Laval, QC, Canada). The ranges of glucose, protein, blood/hemoglobin, specific gravity, pH, Leukocytes, nitrite, ketones, urobilinogen, and bilirubin concentrations were determined semi-quantitatively as described previously [13,14,16].

### 2.4. Immunohistochemistry and Western Blotting Analyses

Urothelial cells and tissues were scratched from freshly removed bladders of UPII-mutant Ha-ras mice and protein lysates were prepared through homogenization as previously described [13,14,16]. Amounts of 10 to 50 μg protein lysates were used for Western blotting analysis of the protein expression of TSC2, phospho-mTOR, 4E-BP1, S6, Skp2, and β-tubulin (as a loading control) using a previously established protocol [10]. All the antibodies were purchased from Cell Signaling Technologies (Danvers, MA, USA).

In addition, proliferating cells on formalin-fixed and paraffin-embedded bladder tissue sections from mice fed with normal drinking water or SHR-5-containing water were determined by IHC analysis using previously reported protocols. Anti-Ki-67 antibody (1:800) was obtained from Abcam an anti-phospho-MAPK (at Thr202/Tyr204) Antibody antibody (1:50), anti-Phospho-S6 Ribosomal Protein (rpS6) (Ser235/236), and an anti-p27/Kip1 antibody from Cell Signaling Technologies (Danvers, MA, USA) [13,14,16]. The quantification of the Ki67-positive cells was carried out by counting the total number of positively stained cells at 5 arbitrarily selected fields at ×200 magnification in a double-blinded manner.

### 2.5. Global Metabolic Analysis

Snap-frozen bladder tumor tissue samples (*n* = 7/group) were obtained from UPII-mutant Ha-ras mice that were fed with normal drinking water or 1.25 or 6.25 mg/mL SHR-5 daily for about five months. The samples were mechanically disaggregated into deionized water and then extracted into methanol. After extraction, the samples were then split into three aliquots for untargeted metabolic profiling and randomized for analysis. Samples were characterized using three independent platforms: ultra-high-performance liquid chromatography–tandem mass spectrometry (UHPLC/MS/MS) in the negative ion mode, UHPLC/ MS/MS in the positive ion mode, and gas chromatography–mass spectrometry (GC/MS) after sialylation. Instrument variability was determined by calculating the median relative standard deviation (RSD) for the internal standards that were added to each sample before injection into the mass spectrometers. Overall process variability was determined by calculating the median RSD for all endogenous metabolites (i.e., non-instrument standards) present in 100% of the Client Matrix samples, which are technical replicates of pooled client samples. The median RSD for instrument and total process variability is about 6% and 13%, respectively.

### 2.6. Statistical Analysis

Prism statistic software was used to compute the mean, standard deviations, and confidence intervals of all quantitative data. Tumor, organ, and body weight comparisons between vehicle control and SHR-5 treatments were accomplished using either analysis of variance (ANOVA) or Student’s t-test, followed by the Bonferroni t-test for multiple comparisons. Survival analysis was performed using the log-rank test, and survival curves were computed by using the product limit method of Kaplan and Meier. All statistical measures were two-sided, and *p*-values <0.05 were considered to be statistically significant.

## 3. Results

### 3.1. SHR-5 Drinking Extends the Life of Male Homozygous UPII-Mutant Ha-Ras Transgenic Mice

We aimed to determine whether the oral administration of SHR-5 to male homozygous UPII-mutant Ha-ras transgenic mice during the full process of carcinogenesis increased the survival of the mice. The male homozygous UPII-mutant Ha-ras transgenic model allows us to study the effect of an agent on the survival (one of the most important endpoints for cancer prevention) of bladder-tumor-bearing mice in a reasonable period [13,14,16]. Therefore, a cohort of male homozygous UPII-mutant Ha-ras mice was genotyped by Southern blotting, then randomized into normal drinking water or water supplemented with 1.25 or 6.25 mg/mL SHR-5, and then sacrificed at 180 days of age. About 33% of the male homozygous UPII-mutant Ha-ras transgenic mice that drank regular water survived over 180 days, whereas more than 95% of male homozygous UPII-mutant Ha-ras mice that drank 1.25 or 6.25 mg/mL SHR-5 survived more than 180 days of age (Figure 1B). SHR-5 drinking markedly increased the survival rate of the transgenic mice by an absolute 65% (Figure 1B, log-rank tests, *ps* < 0.0001). There is almost no difference in survival rate between low and high doses of SHR-5 (95% vs 95.8%). SHR-5 drinking for about 180 days did not significantly affect body weight gain over time (Figure 1C) or the weights of the lungs, liver, spleen, heart, or thymus (Figure 1D). However, SHR-5 drinking at a concentration of 6.25 mg/mL significantly decreased the prostate weight of the mice (*p* < 0.05, Figure 1D). However, there are no significant changes in histological morphology in different organs, including the liver, spleen, kidney, lungs, etc. (Supplementary Figure S1).

### 3.2. SHR-5 Drinking Reduces the Weight of Tumor-Bearing Bladders

**Figure 2:**
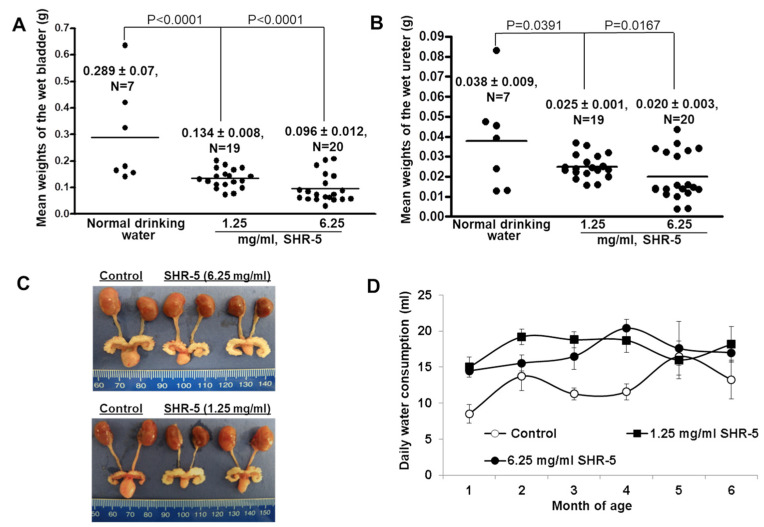
The effect of SHR-5 drinking on bladder and ureter weights of UPII-mutant Ha-ras transgenic mice. (**A**,**B**) Bladder and ureter weights of male homozygous UPII-mutant Ha-ras transgenic mice that were fed with regular water or water containing 1.25 or 6.25 mg/mL SHR-5 until 180 days of age. (**C**) Macroscopical examination of bladders, ureters, and kidneys after the transgenic mice were fed with regular water or water containing 1.25 or 6.25 mg/mL SHR-5 until 180 days of age. (**D**) Average daily water consumption of the mice in indicated treatment groups over time. Error bars represent standard deviation.

Figure 2A shows that SHR-5 drinking at 1.25 and 6.25 mg/mL concentrations both significantly decreased the mean bladder weight, which was used as a surrogate for tumor growth, of surviving mutant Ha-ras mice by about 54 and 67% at 180 days of age, respectively (*ps* < 0.0001). The same concentrations of SHR-5 drinking also reduced the mean weights of the ureter by approximately 44% and 47% (Figure 2B), which appears to be less effective. The gross anatomy of the urogenital system in these mice revealed larger sizes of kidneys, ureters, and bladders, and the transgenic mice that drank SHR-5 exhibited smaller-sized bladders, ureters, and kidneys (Figure 2C). The mice appear to drink more SHR-5-containing water during the first four months of age compared to pure water (Figure 2D).

### 3.3. SHR-5 Drinking Blocks the Progression to Papillary Carcinoma in the UPII-Mutant Ha-Ras Mice

**Figure 3:**
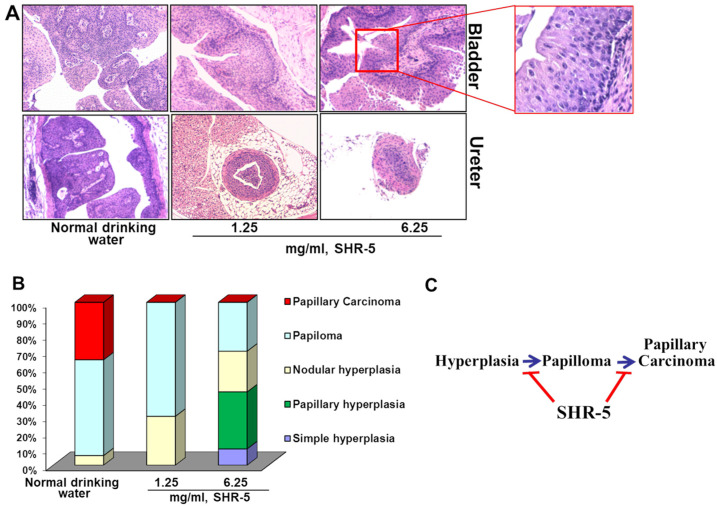
The effect of SHR-5 drinking on pathological progression from hyperplasia to papilloma and UCC. (**A**) H&E images of bladders and ureters from the male UPII-mutant Ha-ras mice after drinking with normal water or SHR-5-containing water for five months. The same control group was used as described in a previously published paper by Liu et al. [13]. Magnification: 100×. (**B**) Percentages of papillary hyperplasia, nodular hyperplasia, papilloma, and papillary carcinoma of the mice in indicated treatment groups. (**C**) Simple graphic presentation of the SHR-5’s effects on papillary UCC development in the male UPII-mutant Ha-ras mice.

Bladders and ureters from the UPII-mutant Ha-ras mice presented with papilloma and papillary carcinoma at 6 months of age (Figure 3A). However, the mice that drank SHR-5, at both 1.25 and 6.25 mg/mL concentrations, showed a complete reduction in papillary carcinoma occurrence (Figure 3A,B). A high dose of SHR-5 also decreased the formation of papilloma by an absolute 30% (Figure 3B, *p* < 0.01).

### 3.4. SHR-5 Drinking Improves Hematuria and Obstructive Uropathy in the UPII-Mutant Ha-Ras Mice

**Figure 4:**
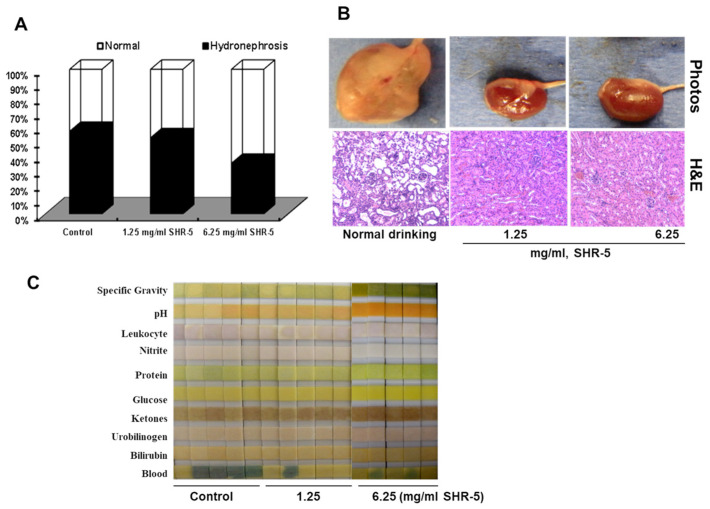
The effects of SHR-5 drinking on uropathy. (**A**) Percentages of mice with hydronephrosis in different treatment groups. (**B**) Six-month-old UPII-Ha-ras mice that drank normal water (control) showed enlarged kidneys, dilated tubular structures, and ureter, whereas age-matched H-ras mice that drank SHR-5 daily showed relatively normal kidneys and no dilation of the ureter. (**C**) SHR-5 drinking decreased blood urine in UPII-mutant Ha-ras mice. Urine was collected from the mutant Ha-ras mice and examined by urinalysis test strips.

The majority (about 60%) of UPII-mutant Ha-ras mice developed hydronephrosis and obstructive ureters, and there was less uropathy in the mice that drank SHR-5 (50 and 30% for 1.25 and 6.25 mg/mL SHR-5, respectively) (Figure 4A,B).

Approximately 80% of the four-month-old UPII-mutant Ha-ras mice that drank normal water exhibited blood urine (>250 erythrocytes/microliter in urine), whereas 20% and 0% of the mice that drank 1.25 mg/mL and 6.25 mg/mL SHR-5, respectively, exhibited blood urine (Figure 4C).

### 3.5. SHR-5 Drinking Inhibits mTOR Signaling Leading to Reduced Cell Proliferation in the UPII-Mutant Ha-Ras Mice

**Figure 5:**
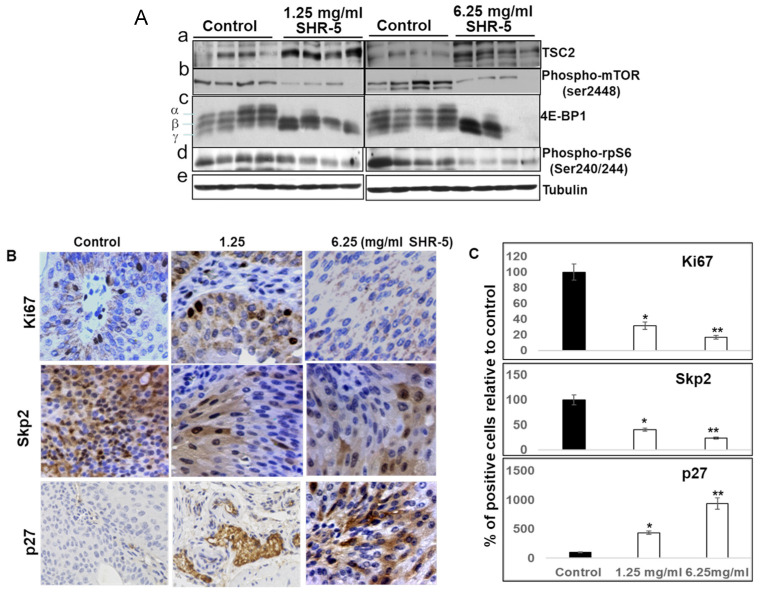
SHR-5 and salidroside inhibit the mTOR pathway. (**A**) Protein lysates were freshly prepared from bladder urothelial tissues from the mice fed with normal drinking water (control), or indicated concentrations of SHR-5, for 5 months. Western blotting analysis was performed to determine the protein levels of TSC2, phospo-mTOR, 4E-BP1, and phosphor-rpS6. Tubulin served as a loading control. (**B**,**C**) IHC-stained tissue sections by anti-Ki67, phospho-MAPK Antibody, and P27 antibodies, respectively, were photographed at ×100 magnifications, and the quantification of percentages of the positively staining cells in each field in treated tumors by normal drinking water, or indicated concentrations of SHR-5, was shown. The same control group was used as described in a previously published paper by Liu et al. [13]. * *p* < 0.05 and ** *p* < 0.01.

Western blotting analysis shows that SHR-5 drinking, at both concentrations of 1.25 mg/mL and 6.25 mg/mL, was associated with markedly increased expression of TSC2 and down-regulated expression of phospho-mTOR (at serine2448), as well as decreased expression of the mTOR down-stream events, phospho-4E-BP1, and Phospho-S6 Ribosomal Protein (rpS6) (Ser235/236) (Figure 5A). Additionally, the mean percentages of Ki67- and phospho-MARK-positive cells per field in the bladder tissues of SHR-5-fed mutant Ha-ras mice were significantly reduced by 68.2% or 59.7% for 1.25 mg/mL SHR-5 and 82.7% or 76.3% for 6.25 mg/mL SHR-5, respectively, compared to the mice drinking normal water (*p* < 0.05 and *p* < 0.01; Figure 5B,C), whereas the percentages of p27-positive cells were significantly increased in the bladder tissues from the mice drinking SHR-5 compared to the control by 3.4- and 8.4-folds for 1.25 mg/mL and 6.26 mg/mL SHR-5, respectively, (*p* values are less than 0.05 and 0.01, respectively, Figure 5B,C).

### 3.6. SHR-5 Drinking Changes Tumor Metabolism in the UPII-Mutant Ha-Ras Mice

From a metabolomics library consisting of more than 2000 standards, a total of 341 named metabolites were detected. A total of 86 (59 up and 27 down) and 50 (47 up and 3 down) metabolites in the bladder tumor tissues of mice drinking 1.25 mg/mL and 6.25 mg/mL SHR-5, respectively, were identified with statistically significant changes compared to those drinking regular water.

**Figure 6:**
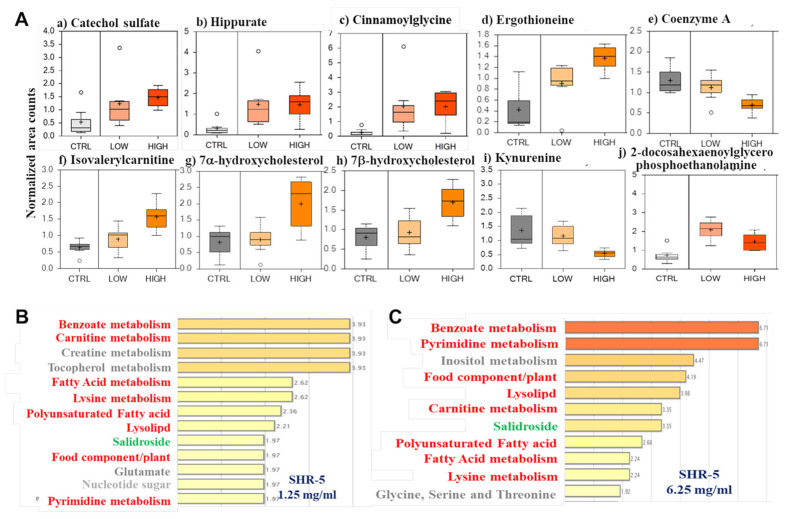
SHR-5 drinking alters tumor metabolism. Global metabolism profiling using UHPLC/MS/MS, in both positive and negative ion mode, and GC/MS methods were performed on bladder tissues from the UPII-mutant Ha-ras mice (*n* = 7/treatment group) drinking normal water or SHR-5-containing water at indicated concentrations for five months. (**A**) Representative metabolites in bladder tumor tissues with dose-dependent and statistically significant alterations by SHR-5 drinking (*ps* < 0.05). The data are presented as a fold change in the SHR-5 drinking relative to the regular water drinking. (**B**,**C**) Significantly enriched metabolic pathways in bladder tumor tissues, which were associated with SHR-5 drinking (*p* ≤ 0.05), are shown.

Phenolic metabolites, including catechol sulfate, hippurate, cinnamoyl glycine, and ergothioneine, were increased in a dose-dependent manner in the SHR-5 treatment groups compared to the control treatment (Figure 6(Aa–Ad)).

Coenzyme A (CoA) is a cofactor required for the metabolism of many substrates including amino acids and fatty acids [17]. The SHR-5-treated samples had reduced levels of CoA when compared with control samples (Figure 6Ae). When CoA is unavailable for the metabolism of acyl chains derived from fatty acids or amino acids, the acyl chain is transferred to carnitine [18]. Several acylcarnitine conjugates were elevated, including isovalerylcarnitine, etc (Figure 6(Af)). The increase in acylcarnitine conjugates by SHR-5 may be due to the decreased level of CoA.

The oxidized metabolites of cholesterol (7-α-hydroxycholesterol and 7-β-hydroxy -cholesterol) were also more abundant in SHR-5-treated samples compared to the control-treated samples (Figure 6(Ag,Ah)), suggesting a disrupted cholesterol homeostasis. Kynurenine, a key metabolite of tryptophan, was also less abundant with the SHR-5 treatment (Figure 6(Ai)). Finally, lysolipids, such as 2-docosahexaenoyl-glycero-phospho- ethanolamine (Figure 6(Aj)), which are generated for membrane remodeling (perhaps as a part of autophagy), were elevated with the SHR-5 treatment.

Pathway enrichment analysis has identified significantly impacted metabolic pathways in SHR-5-treated bladder tumor samples, which include benzoate, carnitine, fatty acid, polyunsaturated fatty acid, lysolipid, food component/plant, lysine, and pyrimidine metabolisms in a dose-dependent manner (Figure 6B,C). Salidroside, a main active component in SHR-5 was also dose-dependently enriched in the SHR-5-treated bladder tumor samples (Figure 6B,C).

## 4. Discussion

More than 500,000 people in the U.S. are bladder cancer survivors [19]. The majority of bladder cancer patients are elderly but may live many years with the disease. During this long-term disease process, bladder cancer patients will experience many mental stresses and possible urological morbidities. *R. rosea* extracts have been shown to prolong health spans in a range of model organisms, including fruit flies, worms, and yeast, up to 31% [20,21,22,23,24,25], to delay the age-related decline of physical activity and immune function, to improve physical and mental performance, and to increase stress resistance [25,26,27]. For the first time, we have shown that male UPII-mutant Ha-ras mice that drink *R. rosea* extract SHR-5 have a markedly high survival rate (more than 95%), reduced tumor burden, and improved uropathy compared to those mice drinking normal water. SHR-5 drinking did not affect body weight gain over time and shows no toxicity in mice. Therefore, our results indicate that SHR-5 deserves further investigation as a “urological care product” to bring a novel impact on bladder cancer prevention and management.

Frequent driver mutations in bladder cancer, including receptor tyrosine kinases, Ras, PTEN, and PI3K, all pathologically activate PI3K/mTOR signaling [28]. The PI3K/mTOR pathway plays a central role in human bladder carcinogenesis [28]. Our previous study has shown that SHR-5, and its main active compound salidroside, treatments resulted in dose-dependent growth inhibitory effects on bladder cancer cell lines with a minimal effect on nonmalignant bladder epithelial cells TEU-2 [10]. SHR-5 and salidroside inhibited mTOR signaling via the activation of AMP-activated protein kinase (AMPK)-α phosphorylation leading to the induction of autophagy [10]. In this study, SHR-5 drinking exerts strong activities on the inhibition of mTOR signaling in vivo in bladder tumor tissues. The UPII-mutant Ha-ras bladder cancer model used here has high mTOR activity, driving tumor growth and progression [13,14]. Thus, this model is a clinically relevant model for studying the inhibitors of the PI3K/mTOR signaling for bladder cancer prevention and treatment. In addition, we have presented evidence from metabolic profiling that drinking SHR-5 is associated with enriched salidroside in bladder tumor tissues in a dose-dependent manner. Taken together, our results suggest that salidroside may contribute to the effects of SHR-5 drinking on the inhibition of mTOR signaling and bladder tumor growth and progression. Further studies are warranted for determining the in vivo anti-tumor effects of salidroside as a pure compound and as an inhibitor of mTOR signaling.

In this study, benzoate and food/plant metabolisms, and kynurenine/serotonin were the top enriched pathways or metabolites by SHR-5. Benzoate is metabolized within mitochondria to produce hippurate [29], and kynurenine and serotonin metabolites were generated by tryptophan catabolism [30]. All these pathways have been linked to host–microbiome signaling [29,30]. Previous studies in flies have shown that the ability of *R. rosea* extracts to prolong lifespan is dependent on diet composition (protein-to-carbohydrate ratios or sucrose contents) at different caloric levels [20,25]. Recent studies have also demonstrated that *R. rosea* root extracts modulate gut microbiota in a Leptin receptor knockout (db/db) mouse model of type 2 diabetes, in BALB/c mice [31], and in flies [32]. Taken together, these results suggest that the observed anti-tumor effects of SHR-5 may be associated with its modulating effects on the gut microbiota. Further experiments are needed to test this hypothesis.

SHR-5 drinking at 6.25 mg/mL significantly increased ketone bodies and decreased pH in urine (Figure 4C). Previous studies have reported the anti-diabetic effects of Rhodiola [31,33,34]. Wang et al. [34] showed that Rhodiola reduced plasma concentrations of non-esterified fatty acids following exogenous glucose stimulation, but that it has minimal effects on sucrose- and olive-oil-induced acute hyperglycemia in animals. This result is consistent with our metabolomic profiling results that Rhodiola drinking significantly affects fat catabolism, which may lead to increased ketone bodies in the urine [35].

In addition, CoA, one of the most critical metabolic cofactors, was dose-dependently reduced in SHR-5-treated bladder tumor tissues. CoA is the major carrier of activated acyl groups in cells and is generated by cysteine, ATP, and vitamin B5 [33]. Very recently, Dibble et al. [36] demonstrated that the de novo synthesis of CoA is regulated by PI3K signaling through AKT substrates pantothenate kinase 2 (PANK2) and PANK4. SHR-5 also exerts concentration-dependent effects on several dipeptides and lysolipids. These effects of SHR-5 may be mediated by its differential effects on the mTOR pathway, which integrates multiple signals (insulin, amino acids, and oxidative stress) to control cell metabolism, growth, survival, and proliferation. Therefore, SHR-5 may inhibit the PI3K/mTOR signaling leading to the reduction in CoA in tumor tissues. Further studies are therefore in progress to determine whether SHR-5 and salidroside affect the expression and activities of PANK2 and 4 through the inhibition of the PI3K/mTOR pathway.

## 5. Conclusions

We have demonstrated the marked chemopreventive efficacy of SHR-5, an anti-aging herb extract, in the UPII-mutant Ha-ras transgenic model of bladder cancer: 95% of the transgenic mice that drank SHR-5 daily, in contrast to 33.3% of the mice in the control group, survived over 6 months of age (*p* < 0.0001). SHR-5 also reduced the occurrences of papillary urothelial cell carcinoma, oncogene-driven metabolism, and growth, and alleviated uropathy. The effects of SHR-5 on tumor metabolisms may be mediated by differential effects on the mTOR pathway. Global metabolomics profiling provides a unique and efficient tool for studying the mechanisms of complex herb extracts’ action.

## Data Availability

Not applicable.

## Supplementary Materials

The following supporting information can be downloaded at: https://www.mdpi.com/article/10.3390/cancers15123086/s1. Figure S1: Microscopical examination of inner organs after SHR-5 or regular water drinking. H&E staining of different organ tissues, magnification 100×.
