# Correction: Liu et al. The Potent Anti-Tumor Effects of Rhodiola Drinking Are Associated with the Inhibition of the mTOR Pathway and Modification of Tumor Metabolism in the UPII-Mutant Ha-Ras Model. *Cancers* 2023, *15*, 3086

**DOI:** 10.3390/cancers17243954

**Published:** 2025-12-11

**Authors:** Zhongbo Liu, Noriko N Yokoyama, Liankun Song, Jun Xie, Zhina Sadeghi, Yi Xi Wu, Sarah Yee, Xue-Ru Wu, Beverly Wang, Edward Uchio, Xiaolin Zi

**Affiliations:** 1 Department of Urology, University of California, Irvine, CA 92868, USA; liuzb727@gmail.com (Z.L.); noriko_yok@hotmail.com (N.N.Y.); liankuns@hs.uci.edu (L.S.); xiej@hs.uci.edu (J.X.); zhinas@hs.uci.edu (Z.S.); yxwu@hs.uci.edu (Y.X.W.); euchio@hs.uci.edu (E.U.); 2 Veterans Affairs New York Harbor Healthcare System, New York, NY 10010, USA; xue-ru.wu@nyulangone.org; 3 Department of Pathology and Laboratory Medicine, University of California, Irvine, CA 92868, USA; bevwang@hs.uci.edu; 4 Chao Family Comprehensive Cancer Center, University of California, Irvine, CA 92868, USA; 5 Veterans Affairs Long Beach Healthcare System, Long Beach, CA 90822, USA

## Error in Figure

In the original publication [1], there was a mistake in Figure 3A as published. Murine bladder and ureter tumor section histology micrographs of control and the SHR-5 (1.25 mg/mL)-treated UPII-mutant Ha-ras mouse in Figure 3A, respectively, were reused or misplaced from the control and Metformin (1 mg/mL)-treated male UPII-mutant H-ras mouse in Figure 2A of Liu-*Mol. Cancer Ther.* **2016**. The corrected Figure 3A appears below. 

In the original publication, there was a mistake in Figure 5B as published. The immunohistochemistry micrographs of the p27 Control and the SHR-5 (1.25 mg/mL) treatment of Figure 5B were reused or misplaced from the control and Metformin (1 mg/mL)-treated male UPII-mutant H-ras mouse in Figure 2A of Liu-*Mol. Cancer Ther.* **2016**, respectively. The corrected Figure 5B,C appears below. 

## Figure Legend

In the original publication, there was missing information in the legend for Figure 3A. The information that the same control group was used as described in Liu et al. *Mol. Cancer Ther.* **2016**, *15*, 430–438. The correct legend appears below. 

The sentence “The same control group was used as described in a previously published paper by Liu et al. [13].” was inserted before “Magnification: 100×.” in the legend for Figure 3A.

In the original publication, there was missing information in the legend for Figure 5B,C. The information that the same control group was used as described in Liu et al. *Mol. Cancer Ther.* **2016**, *15*, 430–438. The correct legend appears below. 

The sentence “The same control group was used as described in a previously published paper by Liu et al. [13].” was inserted before “* *p* < 0.05 and ** *p* < 0.01.” in the legend for Figure 5B,C.

## Text

The sentences “the mean percentages of Ki67- and phospho-MARK-positive cells per field in the bladder tissues of SHR-5-fed mutant Ha-ras mice were significantly reduced compared to the mice drinking normal water (control vs. 1.25 mg/mL or 6.25 mg/mL SHR-5 for Ki67 and phospho-MAPK are 51.3 ± 10.2% vs. 16.3 ± 4.7% or 8.9 ± 2.4%, and 43.4 ± 10.2% vs. 17.5 ± 3.2% or 10.3 ± 2.1%, respectively; *p* < 0.05 and *p* < 0.01; Figure 5B,C), whereas the percentages of p27-positive cells were significantly increased in the bladder tissues from the mice drinking SHR-5 compared to the control (4.5 ± 0.7% for normal drinking water vs. 19.7 ± 3.1% or 42.1 ± 9.6% for 1.25 mg/mL and 6.26 mg/mL SHR-5, respectively. *p* values are less than 0.05 and 0.01, respectively, Figure 5B,C).” were replaced with the following sentences.

“the mean percentages of Ki67- and phospho-MARK-positive cells per field in the bladder tissues of SHR-5-fed mutant Ha-ras mice were significantly reduced by 68.2% or 59.7% for 1.25 mg/mL SHR-5 and 82.7% or 76.3% for 6.25 mg/mL SHR-5, respectively, compared to the mice drinking normal water (*p* < 0.05 and *p* < 0.01; Figure 5B,C), whereas the percentages of p27-positive cells were significantly increased in the bladder tissues from the mice drinking SHR-5 compared to the control by 3.4- and 8.4-folds for 1.25 mg/mL and 6.26 mg/mL SHR-5, respectively, (*p* values are less than 0.05 and 0.01, respectively, Figure 5B,C).”

The authors apologize for any inconvenience caused and state that the scientific conclusions are unaffected, and all new data are from original experimental records and correct. This correction was approved by the Academic Editor. The original publication has also been updated.

## Figures and Tables

**Figure 3:**
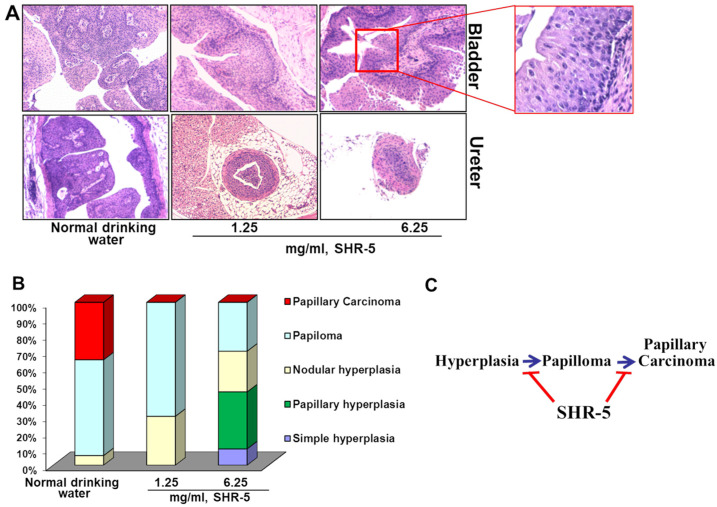
The effect of SHR-5 drinking on pathological progression from hyperplasia to papilloma and UCC. (**A**) H&E images of bladders and ureters from the male UPII-mutant Ha-ras mice after drinking with normal water or SHR-5-containing water for five months. The same control group was used as described in a previously published paper by Liu et al. [13]. Magnification: 100×. (**B**) Percentages of papillary hyperplasia, nodular hyperplasia, papilloma, and papillary carcinoma of the mice in indicated treatment groups. (**C**) Simple graphic presentation of the SHR-5’s effects on papillary UCC development in the male UPII-mutant Ha-ras mice.

**Figure 5:**
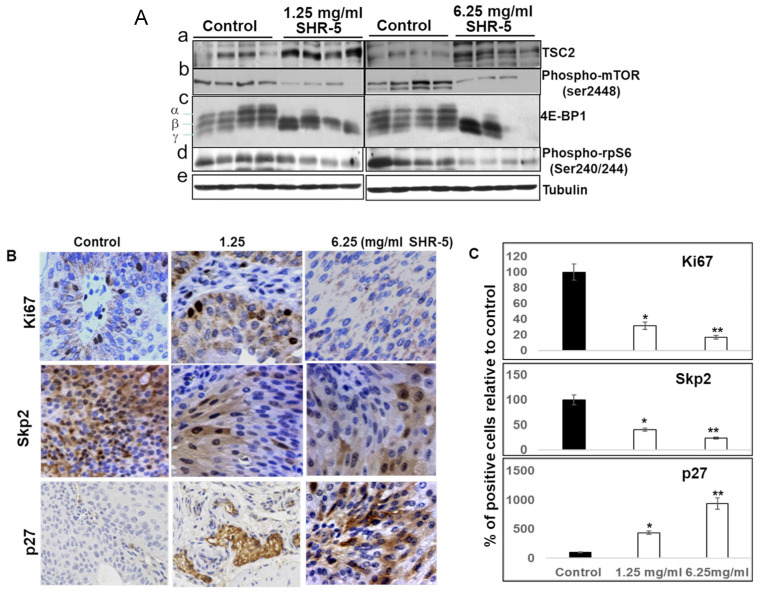
SHR-5 and salidroside inhibit the mTOR pathway. (**A**) Protein lysates were freshly prepared from bladder urothelial tissues from the mice fed with normal drinking water (control), or indicated concentrations of SHR-5, for 5 months. Western blotting analysis was performed to determine the protein levels of TSC2, phospo-mTOR, 4E-BP1, and phosphor-rpS6. Tubulin served as a loading control. (**B**,**C**) IHC-stained tissue sections by anti-Ki67, phospho-MAPK Antibody, and P27 antibodies, respectively, were photographed at ×100 magnifications, and the quantification of percentages of the positively staining cells in each field in treated tumors by normal drinking water, or indicated concentrations of SHR-5, was shown. The same control group was used as described in a previously published paper by Liu et al. [13]. * *p* < 0.05 and ** *p* < 0.01.
