# Supplementary material for: The Potent Anti-Tumor Effects of Rhodiola Drinking Are Associated with the Inhibition of the mTOR Pathway and Modification of Tumor Metabolism in the UPII-Mutant Ha-Ras Model
Source: Cancers (Basel). 2023 Jun 7;15(12):3086. doi: 10.3390/cancers15123086 (PMC10296277; doi:10.3390/cancers15123086)

Supplementary Figure S1. Microscopical examination of inner organs after SHR-5 or regular water drinking. H&E staining of different organ tissues, magnification 100×.

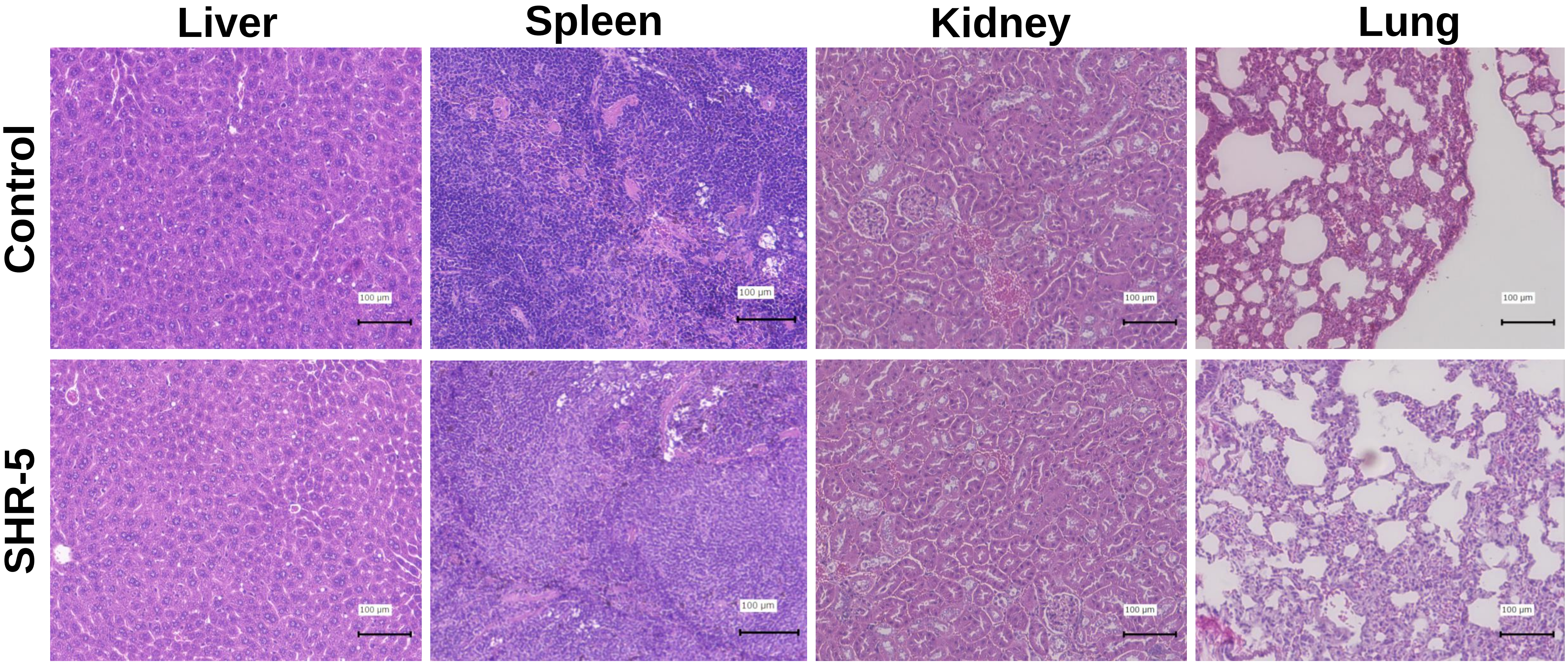

Supplement: Supplementary file 1 [file cancers-15-03086-s001.zip › cancers-2404923-supplementary.pdf]
